# Temporal response of ectopic activity in guinea pig ventricular myocardium in response to isoproterenol and acetylcholine

**DOI:** 10.3389/fphys.2015.00278

**Published:** 2015-10-20

**Authors:** Amara Greer-Short, Steven Poelzing

**Affiliations:** ^1^Center for Heart and Regenerative Medicine, Virginia Tech Carilion Research Institute, Virginia Polytechnic Institute and State UniversityRoanoke, VA, USA; ^2^School of Biomedical Engineering and Sciences, Department of Biomedical Engineering and Mechanics, Virginia Polytechnic Institute and State UniversityBlacksburg, VA, USA

**Keywords:** ectopic, β adrenergic, muscarinic, autonomic, arrhythmia

## Abstract

Both β adrenergic and muscarinic receptor stimulation independently potentiate arrhythmogenesis. However, the effect of simultaneous stimulation on arrhythmogenesis is not well known. The purpose of this study was to determine the temporal response of arrhythmia risk to individual and combined autonomic agonists. Guinea pig hearts were excised and Langendorff-perfused. The β adrenergic receptor and muscarinic receptor agonists were isoproterenol (ISO, 0.6 μM) and acetylcholine (ACh, 10 μM), respectively. All measurements with agonists occurred over 21 min. ISO induced ectopic activity for the first 8 min. ISO also transiently shortened and then prolonged R-R interval over a similar time course. ACh added after ISO transiently induced ectopic activity for 12 min, while R-R interval invariantly prolonged. ACh alone produced few ectopic beats, while invariantly prolonging R-R interval. In contrast to ISO alone, ISO following ACh significantly increased ectopic activity and shortened R-R interval for the duration of the experiment. Animals aged 17–19 months exhibited sustained arrhythmogenesis while those aged 11–14 did not. When ACh was removed in older hearts while ISO perfused, a transient increase in ectopic activity and decreased R-R interval was observed, similar to ISO alone. These data suggest that pre-treating with and maintaining ACh perfusion can sustain ISO sensitivity, in contrast to ISO perfusion alone.

## Introduction

Sympathetic stimulation by β adrenergic receptor (β-AR) agonists, such as isoproterenol or noradrenaline, or by direct stimulation of the stellate ganglion have all been previously used to induce ventricular arrhythmias or precursors to arrhythmias such as spontaneous calcium releases (Opthof et al., 1993; Brodde and Michel, 1999; Xiang, 2011; Lee et al., 2012; Myles et al., 2012). Parasympathetic stimulation via vagus nerves or muscarinic receptor agonists has previously been demonstrated to have opposing effects on calcium handling in myocytes (Gilmour and Zipes, 1985; Nagata et al., 2000), but has also been linked to increase risk of arrhythmias (He et al., 2013). Both β–AR and muscarinic receptor stimulation can *independently* modify cardiac electrophysiology and calcium handling within seconds. Furthermore, prolonged exposure to agonists (minutes to hours) can also modulate responsiveness (Dessy et al., 2000; Obayashi et al., 2006; Whalen et al., 2007; Liu et al., 2012). Therefore, arrhythmogenic risk due to independent β–AR or muscarinic receptor agonists exhibits multiple time scale dependencies.

On the other hand, there is evidence that *simultaneous* stimulation of sympathetic and parasympathetic pathways, either through receptor agonists or by direct nerve stimulation, can produce time dependent effects on heart rate and contractility. Previous studies have demonstrated that the effect of muscarinic receptor stimulation on heart rate and inotropy can be modulated by β–AR stimulation (Hollenberg et al., 1965; Morady et al., 1988; Stramba-Badiale et al., 1991), and this is commonly known as accentuated antagonism. Accentuated antagonism is dependent on the length of exposure to β adrenergic and muscarinic stimulation and the order in which these responses are activated (Yang and Levy, 1992). Nevertheless, the time- and order-dependent effects of β adrenergic and muscarinic agonists on the risk of arrhythmic events is not clear. Furthermore, age is an important determinant of sympathetic-induced responses, as it has previously been shown that the arrhythmogenic propensity during exercise increases with age (Fleg and Lakatta, 1984; Mayuga et al., 1996). However, the effect of aging on arrhythmogenic risk during simultaneous stimulation is not well understood either. Therefore, the purposes of this study are to determine how activating one pathway by direct perfusion of an agonist individually and in combination temporally modulates arrhythmia risk, and how age affects this response.

## Materials and methods

The investigation conforms to the *Guide for the Care and Use of Laboratory Animals* published by the US National Institutes of Health (NIH Publication No. 85-23, revised 1996) and has been approved by Institutional Animal Care and Use Committee (IACUC) at Virginia Polytechnic Institute& State University.

### Experimental preparations

Retired breeder male guinea pigs (ages 11–19 months, weight 800–1200 g, *n* = 45) were anesthetized with sodium pentobarbital (325 mg/kg) or isoflurane inhalation. Hearts were rapidly excised, atria removed, and ventricles were Langendorff-perfused with oxygenated modified Tyrode solution (in mM, CaCl_2_ 1.25, NaCl 140, KCl 4.56, dextrose 5.5, MgCl_2_ 0.7, HEPES 10; 5.5 mL of NaOH used to pH to 7.4) at 37°C and 50 mmHg. Experimental preparation time, or the time from start of surgery to Langendorff-perfusion with Tyrode, was ~5 min. Motion was reduced using 20 μM blebbistatin recirculated for 10 min.

### Arrhythmia induction protocol

**Figure 1:**
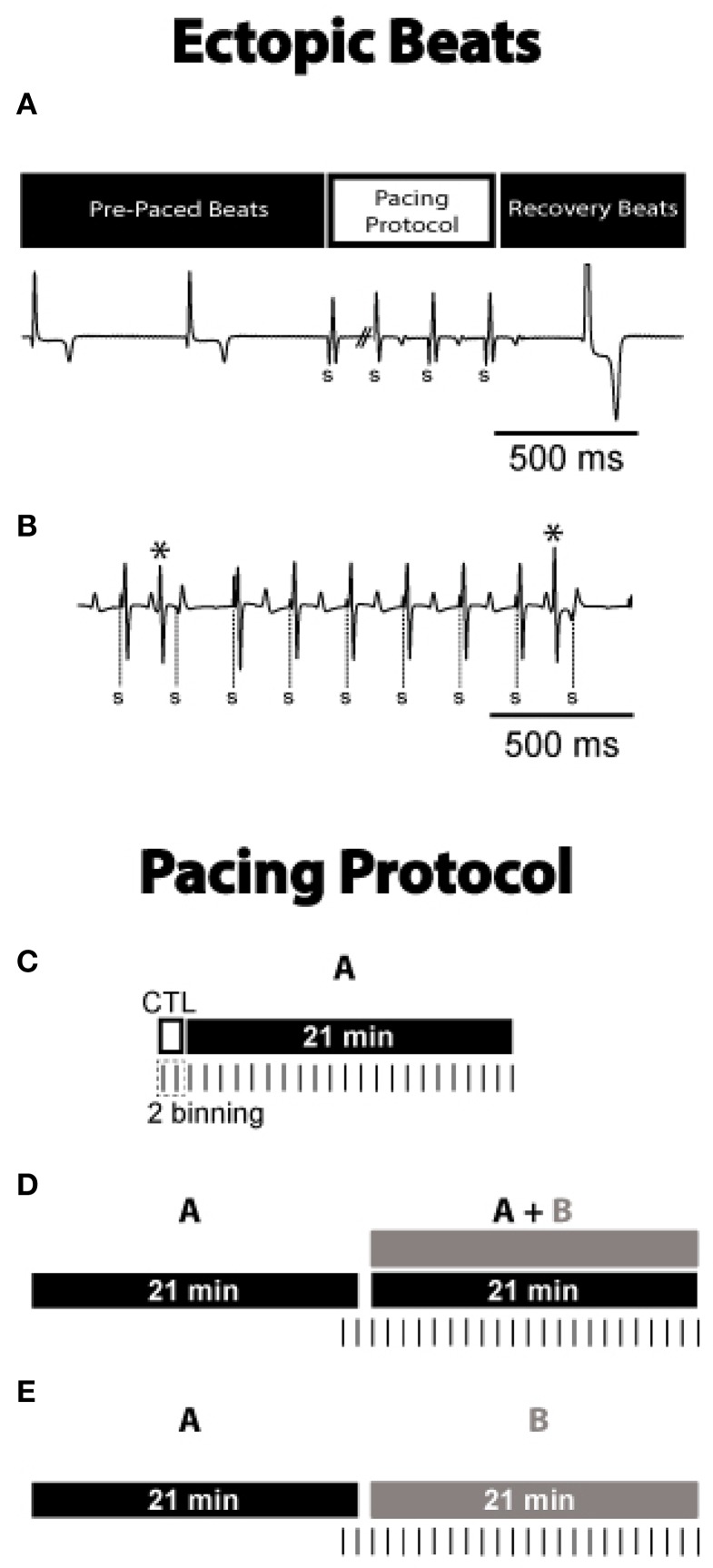
**Ectopic beats during and after pacing. (A)** Representative ECG from ACh + ISO experiment. The first recovery beat following 15 s of rapid pacing and the preceding pre-paced beats are shown. Paced beats are denoted by “S.” Latency and QRS width of the first recovery beat were normalized to the R-R interval and QRS width of the pre-paced beats to determine which beats were ectopic. Displayed is an example of an ectopic recovery beat. **(B)** Representative ECG from ACh + ISO experiment. The rapid pacing drive train is shown with premature beats (*), marked as ectopic beats. **Pacing Protocol**. **(C)** Control conditions without autonomic agonists, were observed for 2 min. ISO or ACh was then perfused for 21 min. Hearts underwent rapid pacing for 15 s every minute and data was binned into 2 min intervals. **(D)** ISO or ACh was perfused for 21 min, and hearts were paced for the last 2 min. The other autonomic agonist was then added to the perfusate for an additional 21 min. **(E)** ISO or ACh was perfused for 21 min with hearts paced for the last 2 min. The solution was then switched to the other autonomic agonist for 21 min.

Ectopic beat burden was assessed using a continuously recorded volume-conducted bath electrocardiogram (ECG). A bipolar plunge electrode in the interventricular septum was used to pace the hearts at 300 bpm for 15 s. The first recovery beat after rapid pacing was evaluated to determine if it displayed ectopic behavior (Figure 1A). A previously established algorithm was used to stratify ectopic beats from other types by normalizing recovery beat latency and QRS width to the corresponding pre-paced intrinsic beat latency and QRS width (Greer-Short and Poelzing, 2015). Premature beats that occurred during pacing were also considered ectopic beats (Figure 1B).

Ectopic beats were counted for 2 min prior to autonomic stimulation (Figure 1C, Control, open bar), and during the subsequent 21 min perfusion of the β adrenergic agonist isoproterenol (ISO, 0.6 μM) or muscarinic agonist (ACh, 10 μM, drug A, black filled bar). Similar ISO doses (0.1–1 μM) have previously been used with guinea pig myocytes to induce early and delayed afterdepolarizations (Yamawake et al., 1992; De Ferrari et al., 1995). ACh physiological concentrations can range from the nanomolar in plasma (Fujii et al., 1995) to the millimolar at the neuromuscular junction (Aidoo and Ward, 2006), and therefore we chose a concentration that has been associated with augmented calcium handling in guinea pig myocytes (Song et al., 1998). In another set of experiments, the autonomic agonist (drug A) was perfused for 21 min, and then was followed by the addition of the other agonist (drug B, gray filled bar, Figure 1D). Figure 1E displays another set of experiments wherein drug A was perfused for 21 min and then followed by the perfusion of just drug B. Vertical dashes below the bars denote when rapid pacing was used to induce ectopic beats. Hearts were rapidly paced every minute for 15 s, and then binned into groups of 2 min (example in Figure 1C). Ectopic beat incidence was calculated by counting the number of pacing protocols that produced ectopic beats and dividing by the total number of pacing protocols performed within the 2 min. The following list reports the sample size (number of animals aged 17–19 months) used for each conditions.

- Control *n* = 3
- +ISO *n* = 6
- ISO + ACh *n* = 7
- +ACh *n* = 4
- ACh + ISO *n* = 13
−ACh + ISO *n* = 5

In a separate set of experiments, animals 11–14 months were studied.

- ACh + ISO *n* = 7. Ages 11–14 Months

### Statistical analysis

Data significance was analyzed using paired and unpaired *t*-tests, Mann-Whitney, and Chi-square where appropriate. A *p* < 0.05 was considered significant. Mean ± standard error was reported.

## Results

### Control

**Figure 2:**
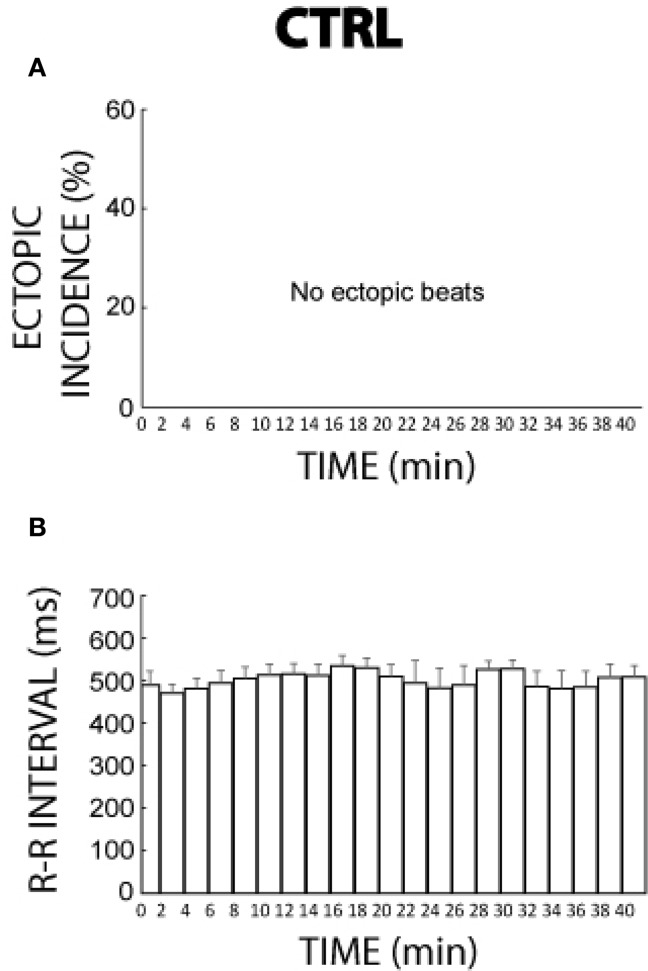
**Control ectopic incidence and R-R interval time course. (A)** No ectopic beats were observed during 41 min of control conditions (*n* = 3). **(B)** R-R interval changed by as much as 29 ms over 41 min without a reproducible temporal pattern.

Without agonists, ectopic beats were never observed over the time course of 41 min either during or following pacing as illustrated in Figure 2A. Figure 2B reveals that over the same time period the R-R interval during control conditions was relatively stable but could vary by as much as 29 ms.

### + isoproterenol

**Figure 3:**
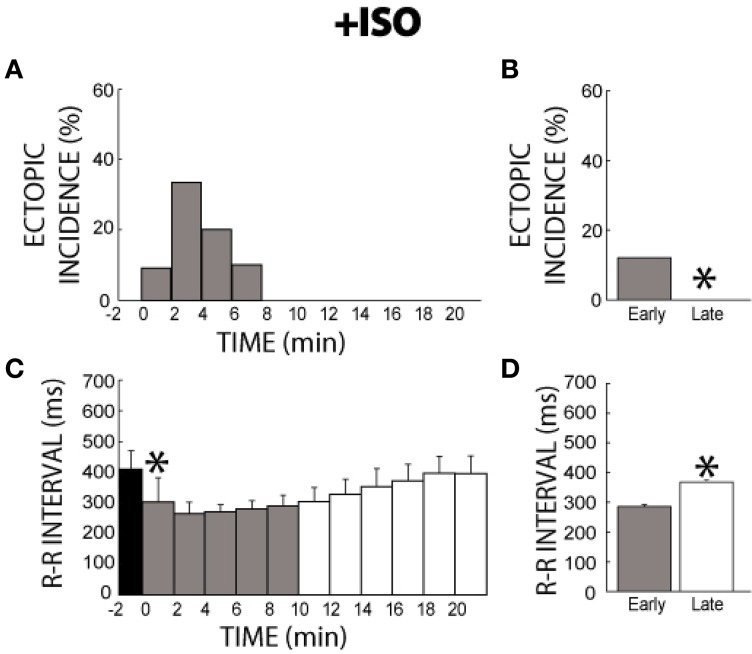
**+ISO ectopic incidence and R-R interval time course. (A)** Without ISO (time −2) no ectopic beats were elicited. With the addition of ISO, ectopic beats were produced for 8 min, reaching the peak incidence by 2 min, before reaching 0% incidence at 8 min. Black: without ISO, Gray: early stage with ISO (0–11 min), and White: late stage with ISO (12–21 min), respectively. **(B)** Significantly more ectopic activity (*) was produced in the early stage (0–11 min, gray bar, 12%, *n* = 6) relative to the late stage (12–21 min, white bar, 0%). **(C)** ISO significantly shortened R-R interval within 2 min (*) relative to time points before ISO perfusion (Black). Over time, R-R interval gradually returned to control cycle lengths. **(D)** The R-R interval for the early stage was significantly shorter relative to the late stage (286 ± 6 vs. 368 ± 8 ms, *).

Before β adrenergic stimulation with ISO, or at −2 min, no ectopic beats were observed (Figure 3A), consistent with the lack of ectopic beats observed during the entire 41 min time-control experiment. After ISO perfusion, ectopic beats were observed, reaching a peak incidence within 2–3 min. By 8 min, ectopic beats were no longer observed. Therefore, a temporal relationship exists for ISO-induced ectopic beats. Quantification of summary data revealed that ectopic beats were significantly more frequent in the early (0–11 min) relative to the late (12–21 min) stage of the experiment (12 vs. 0%, gray bar vs. white bar, Figure 3B), and therefore ISO *transiently* increases arrhythmia risk in the first minutes of stimulation.

Likewise upon administration of ISO, R-R interval significantly decreased within 2 min and then slowly recovered to control rates (Figure 3C). Summary data revealed that R-R interval in the early stage of the experiment was significantly shorter than in the late stage (286 ± 6 vs. 368 ± 8 ms, Figure 3D). Furthermore, this change in R-R interval was significantly larger than R-R variation over 41 min of control conditions (84 ± 11 vs. 29 ± 8), indicating that this change was due to an ISO-induced effect rather than intrinsic changes in Langendorff-perfused guinea pig hearts. Notably, these results suggest that β-agonist responsiveness and arrhythmia risk acutely increases within a few minutes and then decreases in *ex vivo* preparations.

### Isoproterenol + acetylcholine

**Figure 4:**
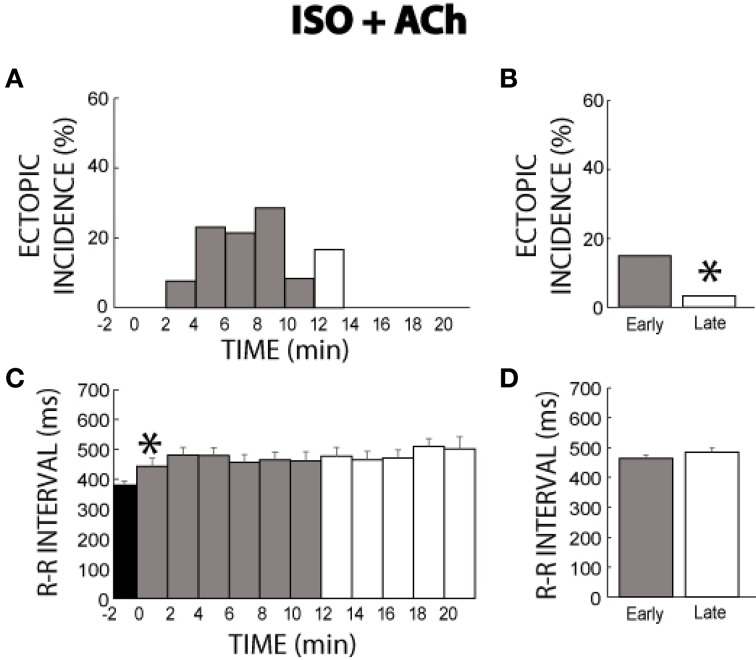
**ISO + ACh ectopic incidence and R-R interval time course. (A)** After 21 min of ISO perfusion (time −2) no ectopic beats were produced. The addition of ACh re-initiates ectopic beats starting at time 2 min and lasts for 12 min before reaching 0% incidence at time 14 min. Black: without ACh, Gray: early stage with ACh (0–11 min), and White: late stage with ACh (12–21 min), respectively. **(B)** Ectopic beat incidence was significantly larger for early (15%, *n* = 7) relative to the late stage (*, 3%), demonstrating a transient temporal relationship. **(C)** ACh prolonged R-R interval within 2 min (*), and cycle length remained elevated for 21 min. **(D)** No significant R-R interval differences were found between the early and late stages (465 ± 11 vs. 485 ± 14 ms).

In a separate set of experiments where ISO was perfused for 21 min and then ACh added to the perfusate, ectopic activity before ACh (time −2 min, Figure 4A) remained at 0% consistent with the last 10 min of the ISO experiments above. Surprisingly, the addition of ACh reinitiated ectopic beats for 12 min. However, the response was *transient*. This is confirmed by the summary data, which revealed more ectopic activity in the early (15%) stage of ISO + ACh than the late stage (3%) as illustrated in Figure 4B.

Data in Figure 4C reveals that unlike ISO alone, ISO + ACh increased the R-R interval immediately upon perfusion and maintained the increased R-R interval for the duration of the experiment. This is supported by summary data in Figure 4D (465 ± 11 early vs. 485 ± 14 ms late).

### + acetylcholine

**Figure 5:**
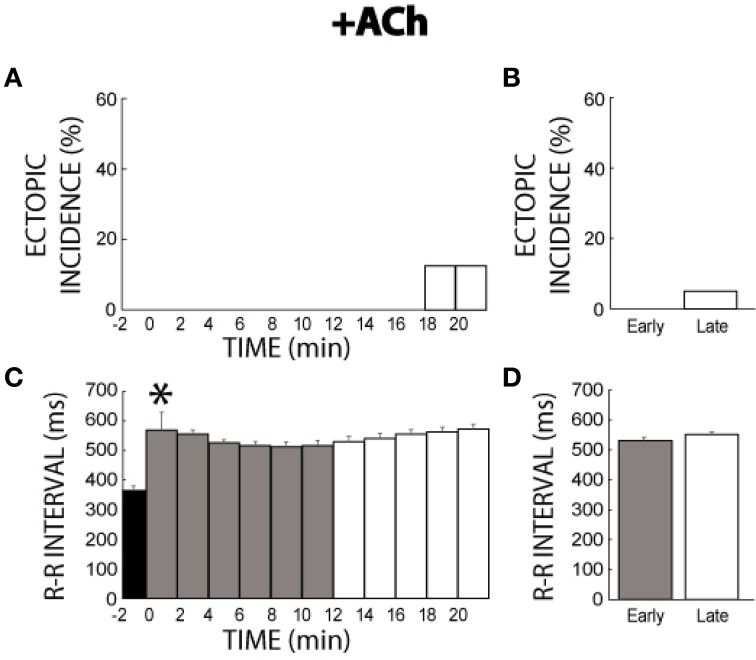
**+ACh ectopic incidence and R-R interval time course. (A)** Ectopic beats were not produced without ACh (time −2). ACh perfusion produced few ectopic beats. **(B)** No significant difference of ectopic activity was found between early (0%, *n* = 4) and late (white bar, 5%) stages. **(C)** ACh increased R-R interval within 2 min (*). **(D)** R-R interval remained prolonged for both the early and late stages (531 ± 10 vs. 551 ± 7 ms).

The muscarinic agonist ACh was not significantly arrhythmogenic. More specifically, only two hearts produced ectopic beats (1 per heart) with ACh between 18 and 21 min (Figure 5A). As a result, ectopic incidence was not significantly different between the early (0%, Figure 5B) and late (5%) measurement stages. ACh increased R-R interval (Figure 5C), and R-R remained prolonged throughout the remainder of the experiment (Figure 5D).

### Acetylcholine + isoproterenol

**Figure 6:**
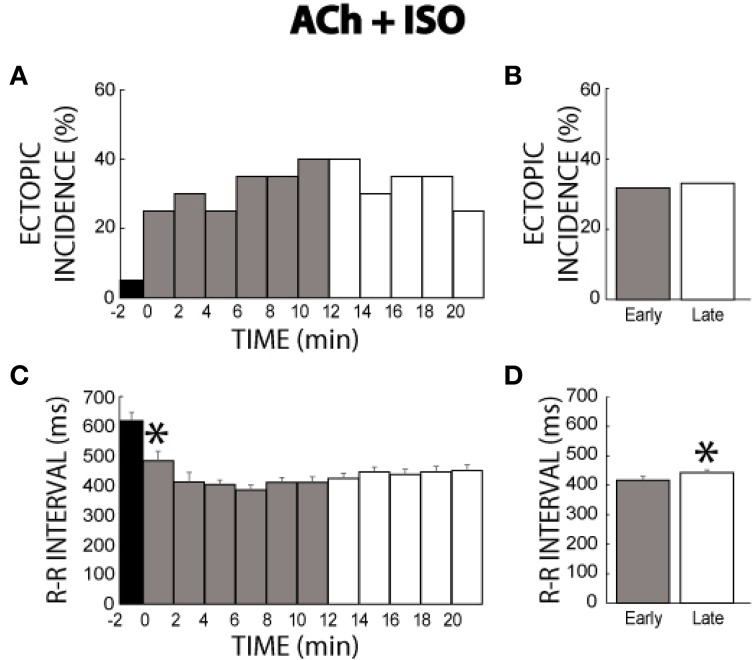
**ACh + ISO ectopic incidence and R-R interval time course. (A)** ACh produced two ectopic beats in one heart (time −2). The addition of ISO elicited more ectopic beats which persisted for the entire perfusion period. **(B)** No significant differences in ectopic incidence were found between the early and late stages (32 vs. 33%, *n* = 10). **(C)** ISO shortened the R-R interval within 2 min (*). **(D)** R-R interval gradually prolonged as evidenced by a significantly shorter R-R interval during the early relative to the late stage (417 ± 10 vs. 442 ± 8 ms, *).

In another set of experiments where ACh was perfused for 21 min before addition of ISO, few ectopic beats were produced (5%, time −2, Figure 6A). The addition of ISO significantly increased the incidence of ectopic beats. Moreover, ectopic beats were *persistently* produced throughout the ACh + ISO perfusion period. Comparisons between the early and late stages revealed that ectopic incidence remained relatively high and unchanged over 21 min (32 vs. 33%, Figure 6B). Importantly, these data demonstrate that pre-treatment with ACh followed by simultaneous ACh + ISO produces a sustained β-adrenergic arrhythmic responsiveness.

Likewise, ACh + ISO decreased R-R interval acutely, but R-R intervals did not return to pre-ISO rates, as happened with ISO alone (compare Figure 6C to Figure 3C). Interestingly, summary data does reveal that early stage R-R intervals (417 ± 10 ms) were significantly shorter than late stage R-R intervals (442 ± 8 ms, Figure 6D). However, while this change was significant, the absolute change in R-R interval was not significantly different to changes observed during control conditions (26 ± 16 vs. 29 ± 11 ms). Furthermore, this change was significantly smaller relative to changes during +ISO (26 ± 16 vs. 84 ± 11 ms), suggesting that pre-treating with ACh followed by simultaneous ACh + ISO blunts β-adrenergic desensitization.

### Acetylcholine + isoproterenol—young guinea pigs

**Figure 7:**
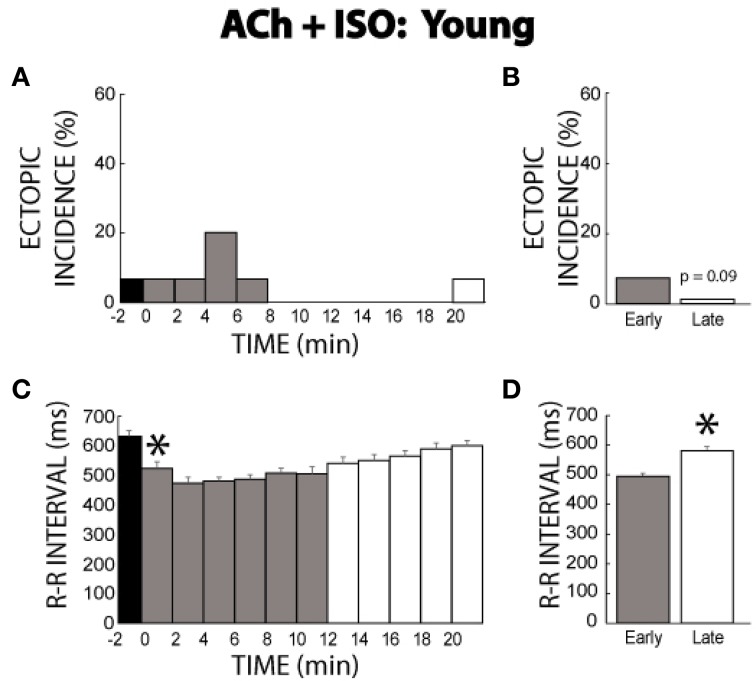
**ACh + ISO ectopic incidence and R-R interval time course in young animals. (A)** One ectopic beat was produced with ACh (time −2). With ISO the ectopic beats were exhibited until 8 min, and only one ectopic beat was produced thereafter. **(B)** The early stage had a 7% ectopic beat incidence, while the late stage had 1% (*n* = 7, *p* = 0.09). **(C)** The R-R interval was shortened by ISO and then gradually prolonged over time. **(D)** The early stage exhibited a shorter R-R interval relative to the late stage (496 ± 8 vs. 568 ± 9 ms, *).

Previous experiments presented in Figures 1–6 were completed with mature guinea pigs ages 17–19 months. In order to account for the effects of age on ectopic incidence, ACh + ISO experiments were performed in guinea pigs ages 11–14 months. Similar to the mature animals, ACh alone produced one ectopic beat. Upon ISO perfusion, ectopic beats were *transiently* produced, with only one ectopic beat observed after 8 min (Figure 7A). The early period ectopic incidence was 7% (Figure 7B), while the late period was 1%. Importantly, young guinea pigs no longer demonstrated persistent arrhythmogenesis.

*Transient* behavior was observed in changes in the R-R interval as well. As expected, ISO decreased the R-R interval, but this was followed by an increase in R-R interval over time (Figure 7C). This was evident in the summary data as well (496 ± 8 early vs. 568 ± 9 ms late, Figure 7D). As would be expected, the change in R-R induced by Ach + ISO was significantly larger compared to control measurements without intervention (72 ± 21 vs. 29 ± 11).

### Heart rate and age effects on ectopic appearance

**Figure 8:**
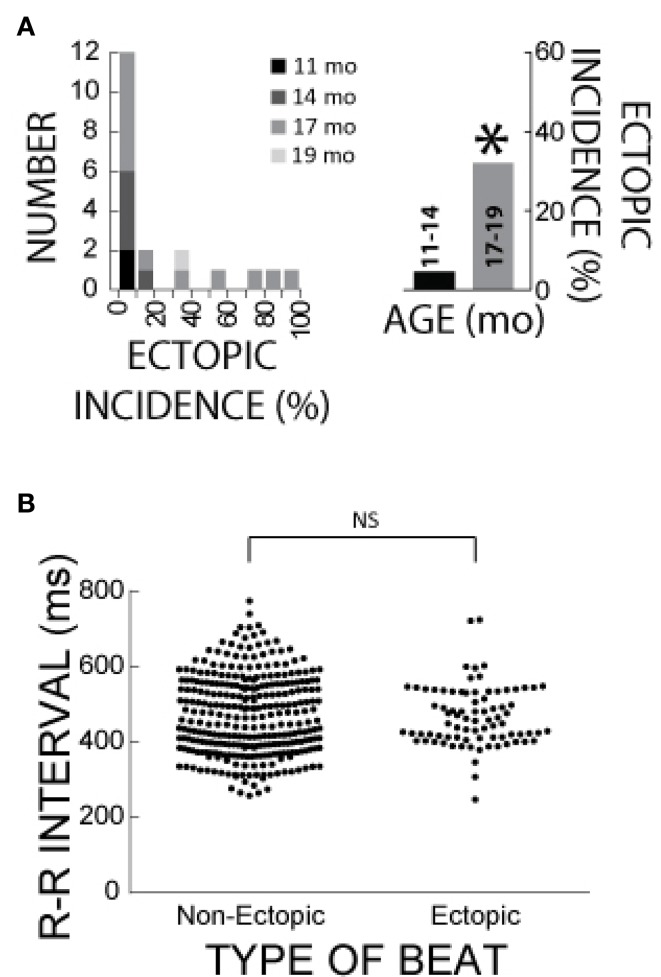
**Age and R-R interval effects on ectopic behavior in ACh + ISO. (A)** Left Panel: Histogram of hearts that manifested differential ectopic beat incidences for the ACh + ISO experiments. Age is represented by the shade of gray. Right panel: Mature guinea pigs (17–19 months) manifested larger ectopic activity incidence relative to young guinea pigs (11–14 months, 31 vs. 5%, *n* = 13 vs. *n* = 7, *). **(B)** The R-R interval of ectopic and non-ectopic beats following ISO perfusion. There was no significant difference in R-R interval.

Figure 8A summarizes the age distribution of the guinea pigs for differential ectopic incidences with ACh + ISO. Importantly, the mature guinea pigs (17–19 months) manifested greater ectopic incidences relative to the young guinea pigs (31 vs. 5%). Total ectopic incidence was defined as the incidence over 21 min of ISO perfusion. This would suggest that age is an important factor in ectopic beat manifestation.

Rapid intrinsic heart rates may also contribute to the trigger of ectopic beats. In order to account for any effects that intrinsic heart rate may have on the formation of ectopic beats, the preceding intrinsic heart rate is compared for the pacing protocol that did or did not produce ectopic beats during ACh + ISO perfusion. There was no significant difference in intrinsic R-R interval between these two groups (Mann-Whitney Test, Figure 8B), suggesting that intrinsic heart rate was not a factor in ectopic beat manifestation.

### −acetylcholine + isoproterenol

**Figure 9:**
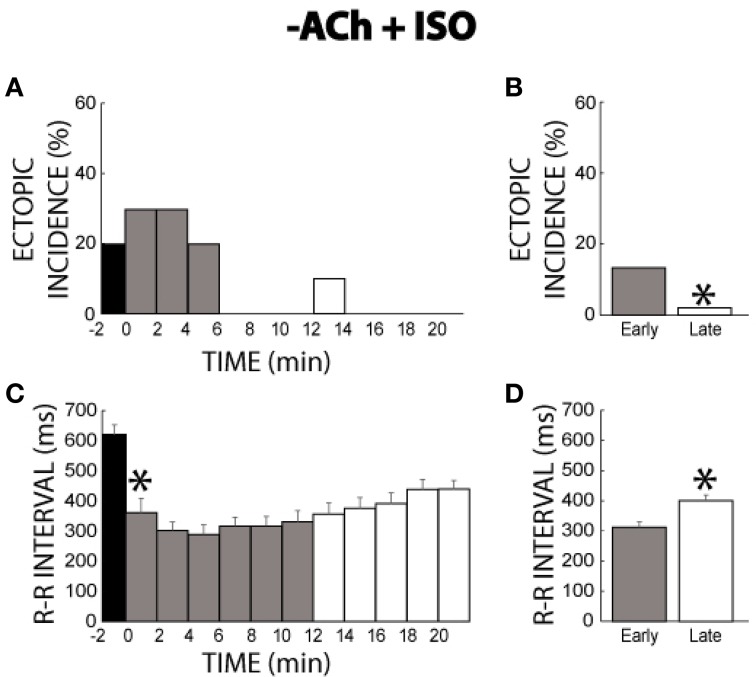
**−ACh + ISO ectopic incidence and R-R interval time course. (A)** Without ISO, ACh produced few ectopic beats. Adding ISO produced ectopic beats for 6 min, but then ectopic activity effectively ceased **(B)** Ectopic beat incidence was significantly larger for early (gray bar, 13%, *n* = 5) relative to the late stage (*, white bar, 2%). **(C)** ISO rapidly shortened the R-R interval within 2 min (*). Over time, the R-R interval gradually prolonged. **(D)** R-R interval was significantly shorter for the early stage relative to the late stage (313 ± 13 vs. 400 ± 15 ms, *).

The previous experiments only explored the concurrent activation of autonomic pathways following chronic activation of the other pathway. However, during normal physiology, parasympathetic tone is usually withdrawn upon activation of sympathetic stimulation. Therefore, we sought to determine whether the persistent behavior observed with ACh + ISO would also be displayed with ACh washout + ISO. As with the initial experiments (Figures 1–6), ACh washout + ISO was performed in mature guinea pigs. Once again, before changing the solutions, 2 ectopic beats were observed from one heart during ACh perfusion (time −2 min, Figure 9A). At time 0, the perfusate with no ACh but with ISO reached the heart. Ectopic beat incidence was elevated for a short time before falling again to a 0% incidence at 6 min. Therefore, withdrawal of ACh and addition of ISO produced a similar *transient* response as ISO alone (compare Figure 9A to Figure 3A). Summary data revealed the same (13% early vs. 2% late, Figure 9B).

Changes in the R-R interval with washout of ACh and wash-in of ISO produced *transient* behavior as well. Specifically, ISO initially shortened R-R interval, but then R-R interval gradually prolonged (Figure 9C). Early and late stage comparisons revealed that the R-R interval was shorter during the early stage (313 ± 13 vs. 400 ± 15 ms, Figure 9D). Importantly, this change in R-R interval was significantly larger than the variations observed under control conditions (88 ± 27 vs. 29 ± 11 ms).

## Discussion

In this study, we demonstrated that continuous pre-perfusion of acetylcholine (ACh) followed by isoproterenol (ISO) creates a persistent arrhythmogenic substrate in mature guinea pigs. ISO and ISO followed by ACh, on the other hand, reveal transient arrhythmogenic substrates. Furthermore, when ACh was washed out during ISO perfusion, the arrhythmogenic substrate became transient again. Therefore, time course and order of β adrenergic and muscarinic receptor stimulation can impact the development of arrhythmias.

### β adrenergic receptor stimulation

#### ISO transiently modifies arrhythmias and R-R interval

ISO perfusion in this study initially increased ectopic activity and heart rate, but this was followed by a gradual decrease in both parameters, suggesting that sympathetic stimulation itself decreases β adrenergic receptor (β-AR) responsiveness. These results are consistent with previous manuscripts that have demonstrated a similar temporal response of heart rate (Joung et al., 2010) and left ventricular diastolic pressure (Yin et al., 2008; *post-hoc* test) during minutes of β-AR stimulation. Consistent with other studies, β-AR stimulation triggers ectopic activity in the whole-heart (Myles et al., 2012; Hoeker et al., 2014). However, to our knowledge this is the first study to demonstrate that *ectopic* activity can decrease within 8 min during ISO perfusion.

The proposed mechanism for this transient response may be attributed to the hypothesis that sympathetic stimulation has a time-dependent, negative feedback mechanism. Specifically, β agonization leads to internalization of β receptors (Liu et al., 2012), desensitization of β2 by switching from coupling with the stimulatory G-protein to the inhibitory G-protein (Whalen et al., 2007) (G_i_), and desensitization of β1 and β2 through activation of phosphodiesterases which degrade cyclic AMP (Nikolaev et al., 2006). Additionally, β3 is also coupled to the G_*i*_ protein. Therefore, during β1 and β2 internalization, stimulation of β3 continues to activate G_i_, further inhibiting global β-AR responsiveness (Curran and Fishman, 1996). As a result of all of these mechanisms, β agonists induce transient and biphasic responses in chronotropy, inotropy, and as we demonstrate, ectopic activity.

### Muscarinic receptor stimulation

#### ACh induces few ectopic beats

Stimulating the muscarinic receptors with ACh led to a sustained decrease in heart rate and few arrhythmias, consistent with previous studies in canine (Farias et al., 2003) and mice (Gehrmann et al., 2002). Parasympathetic stimulation has been associated with arrhythmogenesis in conditions such as long QT syndrome (Fabritz et al., 2010; Shen and Zipes, 2014) and Brugada syndrome (Kasanuki et al., 1997). Generally, however, muscarinic receptor stimulation alone is not arrhythmogenic (Zhang and Mazgalev, 2011), and therefore our results are consistent with previous work.

### Muscarinic following β adrenergic receptor stimulation

#### ACh following ISO reintroduces ectopic beats, but they do not last

Surprisingly, ACh following ISO induced a transient increase in ectopic activity. This is in seeming contradiction to a different study in isolated guinea pig myocytes (Song et al., 1998), as Song et al. found that ACh decreased the amplitude of ISO-induced delayed after-depolarizations, and therefore seemingly displayed anti-arrhythmic behavior. These studies, however, may not be directly comparable because the Song et al. study evaluated the effect of ACh remodeling after only ~5 min of ISO perfusion, and with a significantly smaller ISO concentration (20 nM vs. 600 nM). We chose to use 600 nM ISO, because it was previously demonstrated to induce phosphorylation of proteins that desensitize β-ARs (Liu et al., 2009).

### β adrenergic following muscarinic receptor stimulation washout

#### Removing ACh restores the transient effects of ISO

The effects of ISO following ACh washout have been extensively studied. When ACh is washed out in the presence of ISO, a so-called rebound effect is observed. Specifically, contractility (Hollenberg et al., 1965; McMorn et al., 1993) and calcium current (Wang and Lipsius, 1995) increase and triggered activity is induced (Song et al., 1998) upon ACh washout in the presence of β-AR stimulation. It has previously been proposed that this may be due to activation of the Gq pathway (Colecraft et al., 1998), which has been linked to augmented chronotropy (Kapoor et al., 2015) and inotropy (Proven et al., 2006). Importantly, the response is transient (over minutes), just as reported here.

### β adrenergic receptor following muscarinic receptor stimulation

#### ISO following ACh persistently sustains arrhythmogenesis and changes in R-R interval

It has been demonstrated that sympathetic stimulation within seconds can elevate heart rate even in the presence of chronic parasympathetic stimulation (Yang and Levy, 1992), consistent with this study. To our knowledge, this is the first demonstration that ISO following ACh *persistently* elevates heart rate and ectopic beat incidence. *Importantly*, the persistent elevation of ectopic beat incidence reveals that ISO perfusion in the presence of chronic ACh perfusion in mature animals is a more robust model for producing sustained ectopic activity.

### Mechanisms of enhanced arrhythmic risk

#### Preventing phosphorylation of β-ARs vital for arrhythmogenesis

If ISO exposure reduces β-AR responsiveness, it is not apparent why the order of ACh and ISO perfusion produces temporally different responses. While there is no direct explanation for the persistent elevation of heart rate and ectopic activity only when ISO follows and is concurrently perfused with ACh, previous studies suggest that this may not be an entirely unexpected result. Specifically, β-AR desensitization can occur via Protein Kinase A activation of phosphodiesterases or Protein Kinase A phosphorylation of the β2 receptor, and/or internalization of β-AR. Pre and persistent ACh treatment may *preserve* β-AR sensitivity by inhibiting cyclic AMP production (see Harvey and Belevych, 2003), thereby reducing Protein Kinase A activation, and preventing β2 desensitization.

It is important to note ACh can also activate PKC, which *enhances* G-protein coupled receptor kinase phosphorylation (Yang and Levy, 1992) and thereby can *increase* β internalization (Limas and Limas, 1985). By increasing internalization, ACh may blunt the β-AR negative feedback mechanism. However, this seems unlikely because if receptors were internalized, then washing out ACh and adding ISO should have had similar effects as ACh and ISO, as the time course of 40–50% β-AR receptor recovery post-internalization has been estimated to be about 20 min in rodent (Limas and Limas, 1984; Li et al., 2013). Despite these intriguing hypotheses, further studies are necessary to determine the mechanisms by which ACh modulates β internalization.

### Age-related effects on β adrenergic receptor following muscarinic receptor stimulation

#### Arrhythmia prevalence increases with age

Ventricular arrhythmia prevalence has previously been shown to increase with age, consistent with our results. In particular, age is an important factor in exercise-induced arrhythmias or during sympathetic stimulation (Fleg and Lakatta, 1984; Mayuga et al., 1996). Structural heart changes (Swinne et al., 1992) or remodeling of calcium handling proteins (Grandy and Howlett, 2006) may account for these changes. However, Cerabai et al. demonstrated that β-AR responsiveness reduces with age, and the authors suggested that this may be due to downregulation of the β1 receptor (Cerbai et al., 1995). Therefore, the β2 receptor may play a larger role in an aged heart. As mentioned above, β2 receptors may play an important part in sustaining arrhythmias under the conditions presented here. This requires further investigation though.

## Limitations

The concentrations of isoproterenol and acetylcholine were chosen to elicit measurable responses in the heart. However, as only one concentration for each autonomic agonist was used in this study, it should be noted that other concentrations may elicit different responses. Finally, it is important to note that this study utilized autonomic agonist perfusion into the heart rather than directly stimulating nerves, and direct nervous stimulation might produce different responses than what has been found here.

## Conclusions

In summary, we present evidence that muscarinic receptor stimulation produces ectopic beats in the presence of β adrenergic receptor (β-AR) stimulation in mature animals. Importantly, the order in which muscarinic and β-AR stimulation is introduced has important transient and persistent effects on ectopic beats and heart rate, and these data support the hypothesis that muscarinic receptor stimulation may be impacting ectopic beat formation by modulating β-AR desensitization. Therefore, future studies may consider chronic ACh perfusion before β-AR agonists in order to elicit more ectopic beats. Furthermore, while beta-blocker therapy may be highly efficacious for preventing sudden cardiac death, these findings suggest that preventing ACh induced preservation of β-adrenergic responsiveness may be a new target for preventing sudden death.

### Conflict of interest statement

The authors declare that the research was conducted in the absence of any commercial or financial relationships that could be construed as a potential conflict of interest.

## Abbreviations

ACh: Acetylcholine
β–AR: β–Adrenergic Receptor
ISO: Isoproterenol.
